# Supplementary material for: Baseline Characterization of the Gut Microbiota of Field and Colony Populations of Phlebotomus tobbi and Preliminary Assessment of the Anti-Leishmanial Activity of Cultivable Bacteria
Source: Pathogens. 2026 Jun 23;15(7):658. doi: 10.3390/pathogens15070658 (PMC13416292; doi:10.3390/pathogens15070658)
Supplement: Supplementary file 1 [file pathogens-15-00658-s001.zip › pathogens-4357168-File S1/Taxa2ASV_Decomposer/Genus/g__Delftia/heatmap_with_clustering.pdf]

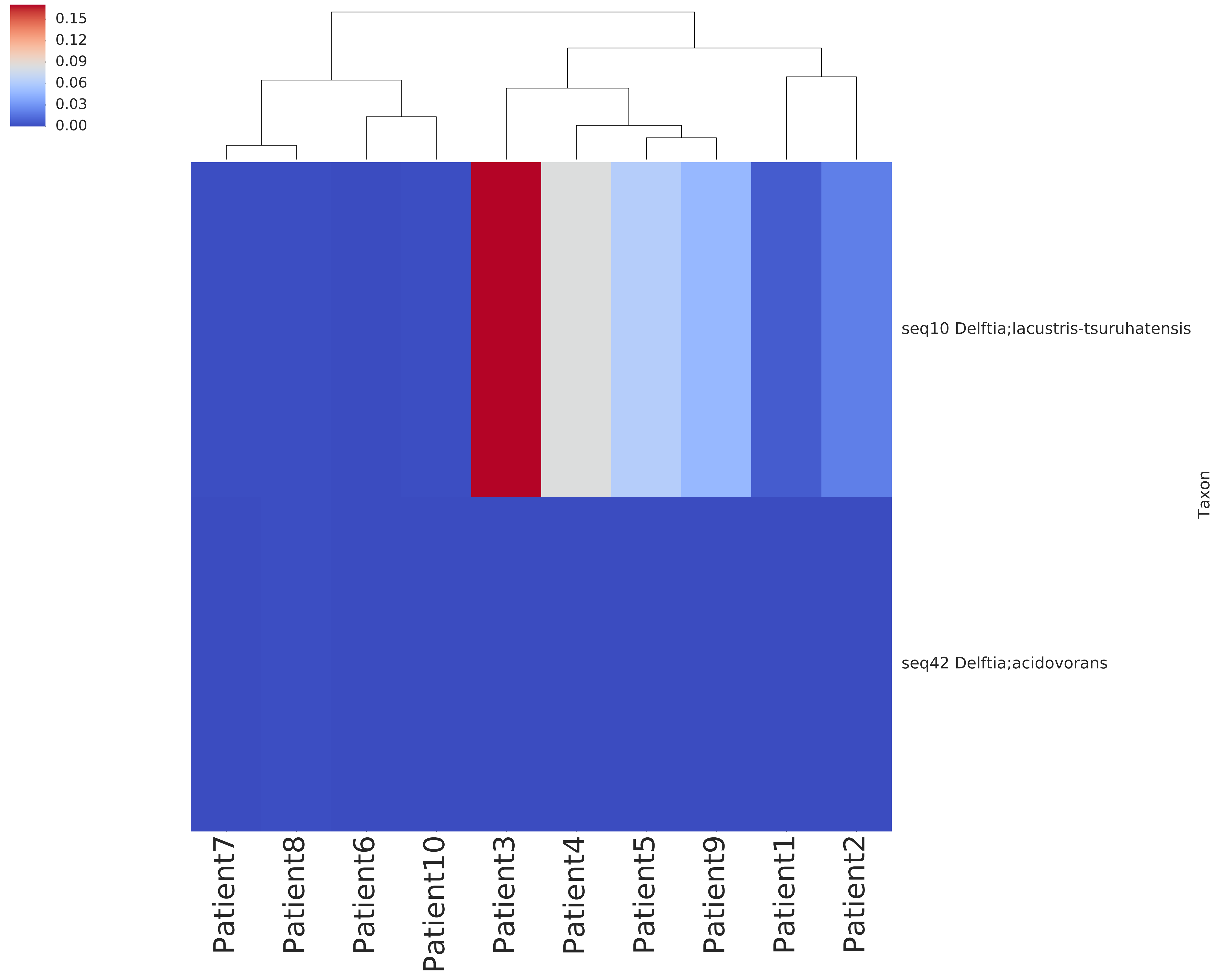
0.15
0.12
0.09
0.06
0.03
0.00
seq10 Delftia;lacustris-tsuruhatensis
seq42 Delftia;acidovorans
Taxon
Patient7
Patient8
Patient6
Patient10
Patient3
Patient4
Patient5
Patient9
Patient1
Patient2
